# Wild-type transthyretin cardiac amyloidosis: the journey to diagnosis in the Czech Republic: The research project of the Czech Society of Cardiology

**DOI:** 10.1186/s13023-026-04249-x

**Published:** 2026-02-13

**Authors:** Barbora Chocholova, Ivana Weislova, Hana Poloczkova, Eniko Marczibal, Renata Aiglova, Milos Kubanek, Jan Krejci, Tomas Palecek

**Affiliations:** 1 2nd Department of Medicine - Department of Cardiovascular Medicine, General University Hospital in Prague, Prague, Czech Republic; 2 Department of Cardiology, Institute of Clinical and Experimental Medicine, Prague, Czech Republic; 3 1st Department of Internal Medicine - Cardioangiology, St. Anne´s University Hospital and Masaryk University, Brno, Czech Republic; 4 Department of Internal Medicine I - Cardiology, University Hospital Olomouc, Olomouc, Czech Republic

**Keywords:** Cardiac amyloidosis, Transthyretin, Heart failure, Arrhythmias, Scintigraphy, Endomyocardial biopsy

## Abstract

**Background and aim of the study:**

Wild-type transthyretin cardiac amyloidosis (ATTRwt-CA) is an increasingly recognized disease in elderly individuals. We aimed to perform a cross-sectional survey investigating the current diagnostic journey of ATTRwt patients in the Czech Republic from initial symptoms to final diagnosis.

**Methods:**

Between November 2022 and May 2023, most data were prospectively obtained using a dedicated questionnaire from 118 ATTRwt-CA patients during the regular outpatient consultation. The rest of the data were taken by attending physicians from medical records.

**Results:**

The mean age of the patients at the time of diagnosis was 77 ± 6 years, 85.6% were males. The time interval from the onset of clinical scenario leading to suspicion of CA to suspected diagnosis was 14±24 months, while the subsequent period to definite ATTRwt-CA diagnosis was 2.5±2.3 months. Heart failure primarily led to the suspicion of CA in 61.9%, followed by suspicious echocardiographic findings (12.7%) and atrial fibrillation or flutter (6.8%). The history of carpal tunnel syndrome was the most common extracardiac manifestation found in 53.9% subjects, with median duration of 99 months. The diagnosis of CA was most often first suspected by outpatient cardiologists (33.1%) and cardiologists working at a cardiocenter (25.4%). At the time of diagnosis, NYHA class I or II was present in 10% and 56% of patients, respectively. The definite ATTRwt-CA diagnosis was made noninvasively in 63% cases.

**Conclusions:**

A relatively long-time delay still exists between the onset of clinical manifestations and suspicion of CA. Nevertheless, once CA is suspected, the definite ATTRwt-CA diagnosis is made early. Although heart failure still represents the most common clinical scenario, several other clinical profiles lead quite frequently to suspicion of the disease. The non-invasive diagnostics of ATTRwt-CA is done in majority of our patients, who are diagnosed mostly in early stages of the disease. The awareness of ATTRwt manifestations must be increased in order to shorten the whole diagnostic process of the disease.

**Supplementary Information:**

The online version contains supplementary material available at 10.1186/s13023-026-04249-x.

## Background

Amyloidosis is a group of systemic disorders caused by misfolded, insoluble proteins that form amyloid fibrils that deposit in various organs. Extracellular deposition of amyloid in tissues affects their function directly through cytotoxic effect and indirectly due to altered anatomic structure and increased stiffness. Currently, more than 35 amyloidogenic proteins are known and represent the basis for the classification of different types of amyloidosis as well for their clinical manifestation, prognosis and therapy. Cardiac involvement plays invariably a significant prognostic role in any type of amyloidosis [1].

In clinical practice, there are three main types of cardiac amyloidosis (CA): light chain (AL) amyloidosis, hereditary transthyretin amyloidosis (ATTRv) and wild-type transthyretin amyloidosis (ATTRwt). Transthyretin CA has been traditionally considered a rare condition. However, advances in non-invasive diagnosis have dramatically increased the number of diagnosed patients especially with ATTRwt which probably represent the most common form of systemic amyloidosis worldwide. Recent data show that ATTRwt prevalence in elderly patients with heart failure with preserved ejection fraction (HFPEF) may be as high as 10–15%. Similarly, ATTRwt is found in 11% of elderly patients with aortic stenosis undergoing transcatheter aortic valve implantation [2]. Importantly, specific therapy that slows or event potentially halts disease progression is already available [3]. The effect of this therapy is most evident in individuals with less advanced disease; therefore, it is absolutely essential to establish the diagnosis of ATTRwt at an early stage.

In several previous studies, the delayed suspicion of ATTRwt and establishing the diagnosis in the relatively late stages of the disease have been described [4, 5]. However, little is known about the length of the diagnostic process of ATTRwt in the current era of non-invasive diagnostics based on scintigraphy using bone-avid tracers together with availability of the disease-modifying therapy and increased awareness of the disease as such. Therefore, the aim of our cross-sectional survey was to investigate the diagnostic journey of ATTRwt patients in the Czech Republic from initial symptoms to final diagnosis.

## Methods

Between November 2022 and May 2023, data were prospectively collected from ATTRwt-CA patients followed in all 4 specialized Czech referral centers for ATTR cardiomyopathy during their regular outpatient visit. In all subjects, the diagnosis of ATTRwt cardiomyopathy was based on the 2021 Position statement of the ESC Working Group on Myocardial and Pericardial Diseases including negative DNA analysis for ATTRv [6].

During the regular outpatient consultation which took place in the above-mentioned time interval, a dedicated questionnaire prepared by investigators was filled by each patient. Questions were focused on patients´ demographics, medical history and the participant experience during the period prior to final ATTRwt diagnosis including his earliest symptoms commonly associated with ATTR. Additionally, patient’s medical records were reviewed by attending physician to complete clinical history, physical examination, ECG, echocardiographic and scintigraphy findings together with blood chemistry parameters (Supplementary material).

All subjects gave their informed consent for inclusion before they participated in the study. The study was conducted in accordance with the Declaration of Helsinki, and the protocol was approved by the Ethics Committees of all involved centers.

## Statistical analysis

Statistical analysis was performed by the Czech Clinical Infrastructure Network (CZECRIN), Masaryk University Brno, Czech Republic. Standard set of descriptive statistics were used to describe the study cohort. Categorical variables are expressed ad numbers and percentages. Continuous variables are presented as mean±standard deviation (SD) and medians (minimum-maximum).

For the analysis of time-to-event data Kaplan-Meier curves were used. Differences between groups were assessed using the log-rank test, with a significance level of 0.05.

To evaluate the relationship between the patient’s health status at the time of diagnosis and the length of the diagnostic process, logistic regression was used. For this analysis, patients were divided into two groups – patients with a confirmed diagnosis within two months since initial suspicion of CA and patients with confirmed diagnosis more than two months after initial suspicion. Selected variables describing the patient’s health status at the time of diagnosis were evaluated in univariable logistic regression models to identify potential factors influencing the time to confirmation of ATTRwt diagnosis. Variables with a p-value lower than 0.1 were then included in a multivariable logistic regression model with stepwise selection. The p-value 0.05 was chosen as the statistical significance level of the multivariable regression. Furthermore, the linear regression was used to assess the length of the diagnostic process and the patient’s condition at the time of diagnosis. In this analysis, the transformed time from initial suspicion to confirmation of ATTRwt diagnosis was modeled, using the natural logarithm transformation. The approach and the statistical significance levels were the same as for logistic regression described above. All statistical analyses were performed using SAS 9.4.

## Results

The analyzed cohort consists of 118 patients with ATTRwt-CA, surveyed in the given time interval (November 2022 and May 2023). Basic sociodemographic data are shown in Table 1. Majority of the participants were males (85.6%). The average age at time of diagnosis ATTRwt-CA was 77.0 years. Most patients lived as couples (67.8%), almost all had at least one child. More than half of participants lived in areas with fewer than 10.000 inhabitants: villages (35.6%) and small towns (16.1.%).

**Table 1:** Sociodemographic data

Characteristics	
Male gender	101 (85.6)
Age at diagnosis (years)	77.0 ± 5.7 78 [61–89]
Living situation	
Alone	33 (28.0)
Couple	80 (67.8)
With siblings	5 (4.2)
Has child/children	115 (97.5)
Living environment	
Prague – capital city	13 (11)
Large city (> 100.000 inhabitants)	13 (11)
Large town (50.000-100.000 inhabitants)	10 (8.5)
Medium town (10.000–50.000 inhabitants)	21 (17.8)
Small town (< 10.000 inhabitants)	19 (16.1)
Village (< 2.000 inhabitants)	42 (35.6)

Values are given as n (%), mean ± standard deviation or median [minimum-maximum]

**Fig. 1:**
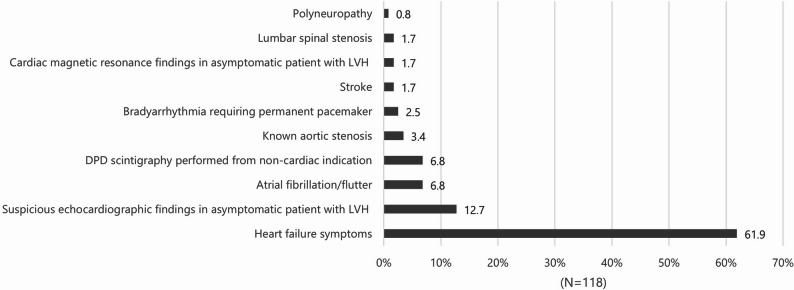
The main clinical profiles leading to suspicion of cardiac amyloidosis (DPD, technetium-99 m-labelled 3,3-diphosphono-1,2-propanodicarboxylic acid; LVH, left ventricular hypertrophy)

The main reasons leading to suspicion of CA are summarized in Fig. 1. Heart failure symptoms were by far the most common clinical scenario (61.9%). The second most common profile represented suspicious echocardiographic findings in an asymptomatic patient with left ventricular hypertrophy (LVH) (12.7%), followed by atrial fibrillation or flutter (6.8%) and abnormal result of technetium-99 m-labelled 3,3-diphosphono-1,2-propanodicarboxylic acid scintigraphy (DPD) performed from non-cardiac indication (6.8%); other clinical scenarios were present less often. In more detail, suspicious echocardiographic findings for CA in the field of LVH were mainly reduced left ventricular (LV) longitudinal function, the presence of Doppler indices of increased LV filling pressures and atrial dilatation. The most common non-cardiac indication for DPD scintigraphy represented staging of prostate cancer.

**Fig. 2:**
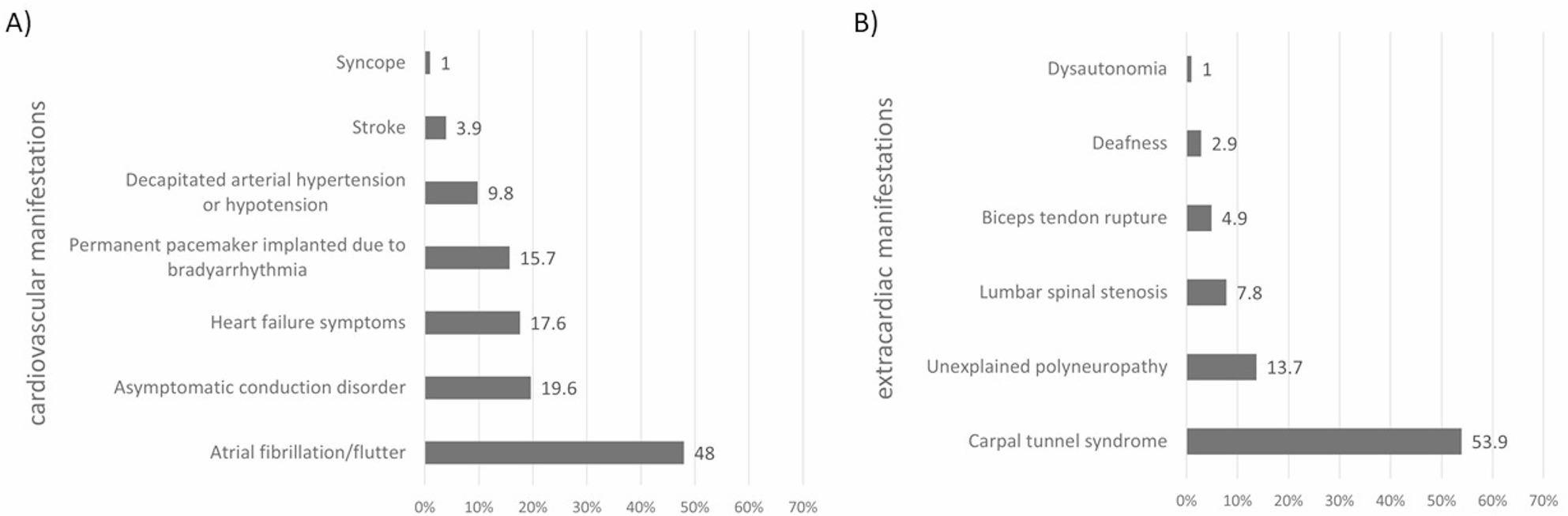
Other typical ATTRwt cardiovascular (part **A**) and extracardiac manifestations (part **B**) already present at the time of suspicion of the disease

Other cardiovascular and extracardiac manifestations possibly related to ATTRwt and already present at the time of suspicion of the disease are given at Fig. 2. Atrial fibrillation or atrial flutter were reported in almost half of the participants (48%), with median duration 36 months (3-201 months). Asymptomatic conduction disorder or permanent pacemaker implanted due to bradyarrhythmia were present in 19.6% and 15.7.%, respectively (medians of duration 48 [7–84] and 39 [1-201] months, respectively). Heart failure symptoms were reported by 17.6% of patients, in whom other reason primarily led to suspicion of CA (median duration 10 [1–60] months). Regarding extracardiac manifestations, the history of carpal tunnel syndrome represented by far the most common feature found in 53.9% individuals, with median duration 99 months (6-372 months). Unexplained polyneuropathy was present in 13.7% (median duration 24 [3-180] months), lumbar spinal stenosis in 7.8% (median duration 24 [4-120] months), and biceps tendon rupture in 4.9% of participants (median duration 96 [12–300] months), respectively.

The diagnosis of ATTRwt-CA was noninvasively established in 74 patients (62.7%). In 44 individuals (37.3%), an endomyocardial biopsy was indicated to confirm the diagnosis. During the diagnostic process, DPD scintigraphy was performed in 115 patients (97.5%); Perugini score 2 was described in 14% and score 3 in 86% of cases, respectively. Cardiac magnetic resonance was done in 46 individuals (39%) during the diagnostic process.

The time interval from the onset of symptoms/clinical scenario leading to suspicion of CA to suspected diagnosis was 14±24 months (median 5.5 months [0-120]. Time interval from suspicion of diagnosis to confirmation of ATTRwt-CA was 2.5±2.3 months (median 2.0 months [0–13]). The total time interval between the appearance of initial symptoms or findings rising the suspicion of CA to final ATTRwt diagnosis was 16.5±24.2 months (median 7.5 months [1-124]). There was no difference in time intervals between the onset of initial symptoms or findings to final ATTRwt diagnosis based on noninvasive examinations or established by endomyocardial biopsy (17.5±26.1 months vs. 14.3±20.0 months, p = NS) and between time intervals from suspicion of diagnosis to noninvasive or invasive confirmation of ATTRwt-CA (2.3±2.1 months vs. 2.8±2.8 months, p = NS).

**Fig. 3:**
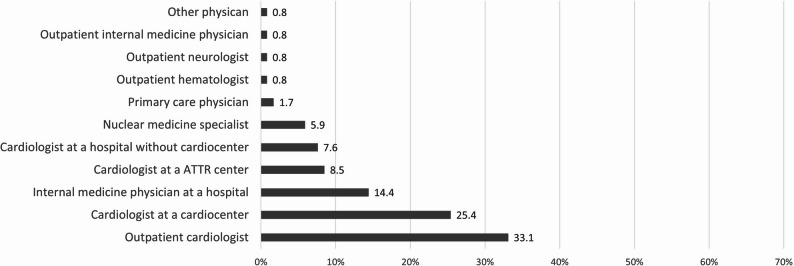
Physicians who first suspected the diagnosis of cardiac amyloidosis (ATTR, transthyretin amyloidosis)

As shown in Fig. 3, the diagnosis of CA was most often first suspected by outpatient cardiologists (33.1%), then cardiologists working at a cardiocenter (25.4%), followed by internal medicine physicians working at a hospital (14.4%), cardiologists based at a center for ATTR (8.5%), cardiologists at a hospital without cardiocenter (7.6%) and nuclear medicine specialists (5.9%), other scenarios were much less common.

**Fig. 4:**
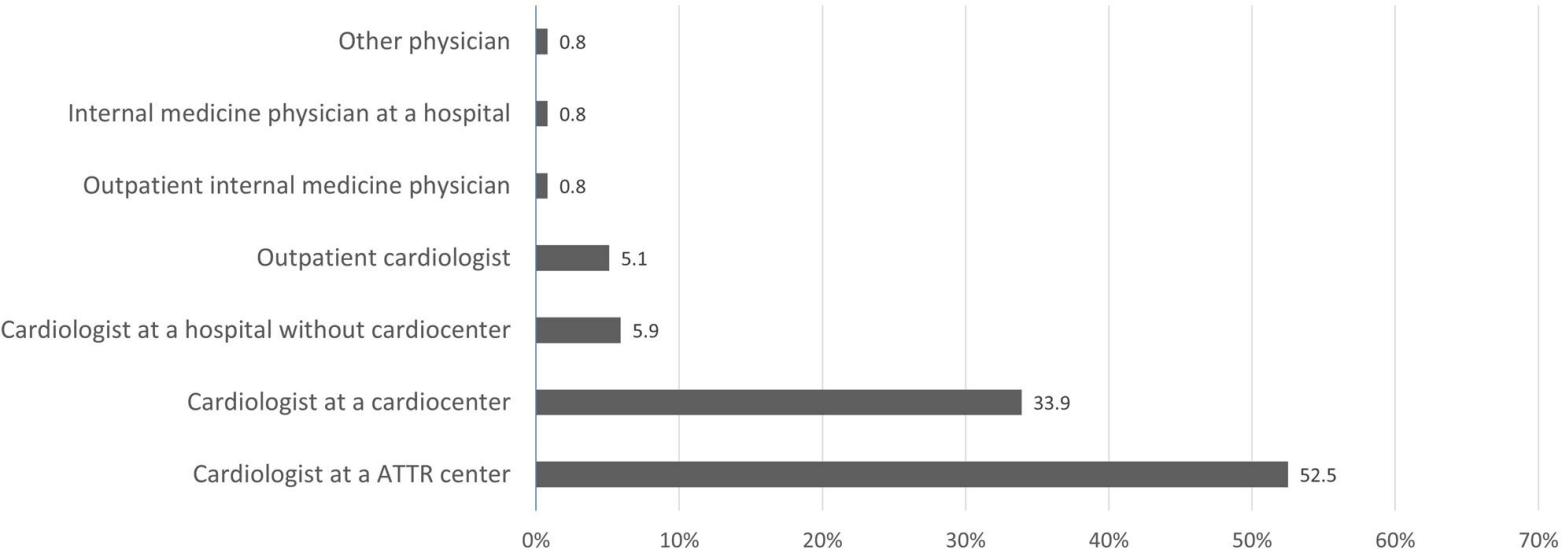
Physicians who made the final ATTRwt-CA diagnosis

The final ATTRwt-CA diagnosis was mostly confirmed by cardiologists based at a center for ATTR (52.5%), followed by cardiologists working at a cardiocenter (33.9%), other physicians made the final diagnosis much less often (Fig. 4).

Since the appearance of symptoms/clinical scenario leading subsequently to suspicion of CA, 44 patients (37.3%) had to be investigated by 2 physicians and 40 individuals (33.9%) by 3 and more physicians until the first suspicion of the disease.

**Fig. 5:**
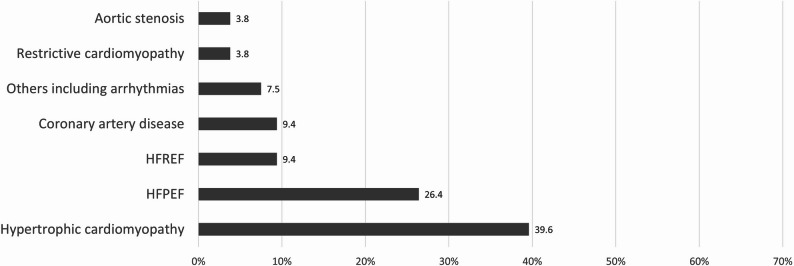
Previous misdiagnoses (HFPEF, hear failure with preserved ejection fraction; HFREF, heart failure with reduced ejection fraction)

Any previous misdiagnosis before CA was suspected was present in 53 individuals (44.9%). Hypertrophic cardiomyopathy represented the most common previous misdiagnosis (39.6%), followed by HFPEF (26.4%), miscellanea of other diagnoses were less common (Fig. 5).

Clinical characteristics of the patients at the time of their first visit to the ATTR center are given in Table 2. Most patients were in NYHA class II (55.9%), then in classes III (30.5%) and I (10.2%); only 4 individuals (3.5%) were in NYHA class IV. There was no significant difference between NYHA classes in terms of serum N-terminal pro B-type natriuretic peptide (NT-proBNP) levels. Regarding the National Amyloidosis Centre (NAC) staging system, 55% of patients were in NAC stage 1, 34% in stage 2 and 11% in stage 3. Diuretic agents, mainly loop diuretics, represented the most commonly used medication (81.4%), followed by angiotensin-converting enzyme inhibitors/angiotensin receptor blockers (67.8%), beta-blockers (62.7%) and mineralocorticoid receptor antagonists (41.5%). Anticoagulants were taken by 60.2% of subjects.

**Table 2:** Clinical characteristics at the time of the first visit to the ATTR center

Characteristics	Total (*n* = 118)
NYHA class	
I	12 (10.2)
II	66 (55.9)
III	36 (30.5)
IV	4 (3.4)
NTproBNP (ng/l)	3050±4014 2035 [179-35000]
eGFR (ml/min/1.73 m^2^)	59±17 58 [29–96]
NAC stage	
1	65 (55)
2	40 (34)
3	13 (11)
Medication	
Diuretics	96 (81.4)
ACEi/ARB	80 (67.8)
ARNI	7 (5.9)
Beta-blockers	74 (62.7)
MRA	49 (41.5)
Calcium channel blockers	14 (11.9)
Anticoagulants	71 (60.2)
Antiarrhythmics Digitalis	9 (7.6) 4 (3.4)

Values are given as n (%), mean ± standard deviation or median [minimum-maximum]

ACEi, angiotensin-converting enzyme inhibitor; ARB, angiotensin receptor blocker; ARNI, angiotensin receptor-neprilysin inhibitor; ATTR, transthyretin amyloidosis; eGFR, estimated glomerular filtration; MRA, mineralocorticoid receptor antagonist; NAC, National Amyloidosis Center; NT-proBNP, N-terminal pro B-type natriuretic peptide; NYHA, New York Heart Association

ECG and echocardiographic data at the time of the first visit to the ATTR center are listed in Table 3. Sinus rhythm was present in 56 patients (46.5%), while atrial fibrillation / atrial flutter or paced rhythm were seen in 43 (36.4%) and 18 (15.3%) individuals, respectively. In 28 subjects (23.7%), first-degree atrioventricular (AV) block was detected. Left anterior fascicular block (LAFB) was present in 28 (23.7%) patients. Right bundle branch block, left bundle branch block and nonspecific intraventricular conduction delay were found in 13 (13.6%), 6 (5.1%) and 10 (8.5%) subjects, respectively. In 14 individuals (11.9%), low QRS voltages were detected, while pseudo-infarct pattern was present in 20 (16.9%) patients. Electrocardiographic criteria for LVH were described only in 2 subjects (1.7%). Regarding echocardiography, asymmetrical septal hypertrophy was found in 6 (5.1%) patients, in all other individuals symmetric LV wall thickening was present. In 37 (31.4%) subjects, global LV systolic dysfunction defined by LV ejection fraction < 50% was described. Increased LV filling pressures defined by peak mitral E wave velocity to peak e´-septal wave velocity ratio > 15, were found in 66 (57.6%) patients. Pulmonary hypertension defined as pulmonary artery systolic pressure ≥ 40mmHg was present in 44 (37.3%) subjects, while increased right atrial pressure was described in 66 (55.9%) patients. Pericardial effusion was seen in 26 (22%) cases.

**Table 3:** ECG and echocardiographic characteristics at the time of the first visit to the ATTR center

Characteristics	Total (*n* = 118)
**ECG**	
Sinus rhythm Atrial fibrillation / atrial flutter Paced rhythm First-degree AV block Second-degree AV block Mobitz I Second-degree AV block Mobitz II Complete AV block LAFB LBBB RBBB Nonspecific IVCD Low QRS voltages /limb leads/ Pseudo-infarct pattern /anterior chest leads/ LVH voltage	56 (47.5) 43 (36.4) 18 (15.3) 28 (23.7) 1 (0.8) 1 (0.8) 1 (0.8) 28 (23.7) 6 (5.1) 13 (13.6) 10 (8.5) 14 (11.9) 20 (16.9) 2 (1.7)
**Echocardiography**	
IVS (mm) LVPW (mm) LVEDD (mm) LVEF (%) s´- septal (cm/s) E/e´-septal LAVi (ml/m^2^) RVEDD (mm) TAPSE (mm) PASP (mmHg)	17.2±3.1 15.1±3.5 46.3±5.7 52.8±9.6 4.8±1.6 17.7±7.2 51.0±13.3 38.7±5.4 17.4±4.2 36.1±12.1
RAP	
normal borderline high could not be estimated Pericardial effusion Aortic stenosis	66 (55.9) 25 (21.2) 25 (21.2) 2 (1.7) 26 (22.0) 14 (11.9)

Values are given as n (%) or as mean ± standard deviation

AV, atrioventricular; ECG, electrocardiogram; E- peak transmitral early diastolic flow velocity; e´- septal, peak early diastolic velocity of the sepal mitral annulus; IVCD, intraventricular conduction delay; IVS, interventricular septal thickness; LAFB left anterior fascicular block; LAVi, left atrial volume index; LBBB, left bundle branch block; LVH, left ventricular hypertrophy; LVEDD, left ventricular end-diastolic diameter; LVEF, left ventricular ejection fraction; LVPW, left ventricular posterior wall thickness; PASP, pulmonic artery systolic pressure; RAP, right atrial pressure; RBBB, right bundle branch block; RVEDD, right ventricular end-diastolic diameter in the apical four-chamber view; s´- septal, peak systolic velocity of the septal mitral annulus; TAPSE, tricuspid annulus peak systolic excursion

Of all clinical, echocardiographic and ECG data, diuretic therapy and female gender were found to be significant predictors of establishing a final ATTRwt-CA diagnosis more than 2 month after initial suspicion in univariate logistic regression model. A multivariate analysis confirmed the prognostic significance both for diuretics (*p* = 0.02, odds ratio 0.23, 95% confidence interval 0.07–0.79) and female gender (*p* = 0.02, odds ratio 0.22, 95% confidence interval 0.06–0.81). However, the odds ratios were associated with very wide confidence intervals, indicating limited precision of the estimates due to the small number of observations in specific exposure–outcome categories. Consequently, diuretic use at the time of diagnosis and female sex cannot be considered robust or clinically meaningful factors influencing the timing or duration of the diagnostic process in ATTRwt-CA. In univariate logistic regression analysis, only NYHA class at the time of the first visit to the ATTR center was found to be a significant factor regarding the time interval from suspicion to confirmation of ATTRwt-CA (*p* = 0.02); however, its significance was not confirmed in multivariate analysis.

## Discussion

Our cross-sectional survey shows several important findings related to the diagnostic journey of patients with ATTRwt in the Czech Republic, which, in our opinion, are of a general importance. A relatively long time period still exists between the appearance of symptoms or clinical scenario that subsequently raise suspicion of CA and final diagnosis of ATTRwt. Although HF symptoms still represent the main scenario leading to suspicion of CA, other clinical profiles also contribute substantially. Several cardiovascular or extracardiac manifestations possibly related to ATTRwt, especially atrial fibrillation and carpal tunnel syndrome, had been already present for a relatively long time before the main diagnostic symptoms or findings appeared. More than third of patients had to visit at least 2 physicians before CA is suspected. Despite this, once CA is suspected, it only takes a short time for it to be confirmed. Most ATTRwt patients are currently diagnosed noninvasively and in less advanced stages of their disease assessed by NYHA classification or NAC stage.

In our survey, the average time from the onset of symptoms or findings which subsequently raised suspicion of CA to definitive ATTRwt diagnosis reached 16.5 months (median 7.5 months). This is similar to the results reported from most other European authors investigating the diagnostic journey of ATTRwt patients in a current era. In the study published by Ladefoged et al., the median diagnostic delay was 13 months (2–47 months) was observed in a unicentric Danish cohort of 50 consecutive patients with ATTRwt diagnosed from 2017 to 2019 [5]. The authors from Toulouse performed a retrospective cross-sectional study on patients with cardiac amyloidosis which included 122 ATTRwt individuals diagnosed between 2001 and 2019 in their centre and reported median delay 10 months (3–34) between symptom onset and final ATTRwt diagnosis [7]. In German AMY-NEEDS Programme substudy, the median diagnostic delay in a prospective cohort of 74 ATTR patients (90.7 ATTRwt) between onset of symptoms and final diagnosis was 12 months (0–127.7) months [8]. The data from the French daily impact of amyloidosis study showed the average time interval of 19.6 months from the initial symptoms until a confirmed diagnosis in 109 ATTRwt patients [9]. For comparison, Lane et al. reported median delay 39 months from first presentation with cardiac symptoms at the cohort of 711 ATTRwt patients referred to NAC (London, Great Britain) between 2000 and 2017 [4]. This difference illustrates considerable improvement in the speed of the whole diagnostic process of ATTRwt-CA in the last years, which is mainly due to greater awareness of the disease together with the improved knowledge of its main manifestations and large availability of non-invasive diagnostic tools, especially scintigraphy using bone-avid tracers. The possibility and access to specific, disease-modifying treatment also plays an important role. However, we still consider diagnostic delay found in our study and reaching an average of 1.5 years to be suboptimal and with the possibility of improvement. As shown in the recent Italian DIAMOND study, overall median time from initial symptoms to diagnosis may last only 4 months [10].

As our data show, the total length of the diagnostic process is primarily determined by the long period between the onset of main symptoms to suspicion of CA, with average duration of 14 months (median 5.5 months). 37% of our patients visited 2 physicians and one third even at least 3 physicians before CA was suspected. This is very similar to recent findings reported by German authors [8]. In their study, 28.8% of symptomatic ATTR patients had received their diagnosis after two contacts with physicians. More than 5 physicians until receiving correct diagnosis had been contacted by 20.3% of symptomatic patients. Almost half of our patients were misdiagnosed with other cardiac diseases. Among them, hypertrophic cardiomyopathy and HFPEF were the most common, representing two thirds of all previous misdiagnoses. In the seminal study published by González-López et al., 35% of ATTRwt patients had been previously misdiagnosed with cardiac disorders. Among them, hypertensive heart disease was the most common, followed by hypertrophic cardiomyopathy, ischaemic heart disease, HFPEF and aortic stenosis [11]. In another study by Spanish authors, the previous diagnosis was obtained in 45% of patients with CA. The most frequent misdiagnosis was again hypertensive heart disease, followed by HFPEF [12]. Ladefoged et al. reported, that 60% of their patients were investigated for at least two non-ATTRwt diagnoses during the time period from the first investigation to the time of the CA diagnosis [5].

On the other hand, or data show that the final diagnosis of ATTRwt-CA is made relatively early once CA is suspected, within 2.5 months on average (median 2 months). This favourable fact is certainly due to the easy availability of scintigraphy throughout the Czech Republic. In 2018, the Czech Society of Cardiology together with the Czech Society of Nuclear Medicine have introduced the specific treatment programme allowing physicians to indicate DPD scintigraphy directly for diagnostics of ATTR-CA.

The first suspicion of CA was raised in slightly more than half of the cases by outpatient cardiologists and cardiologists working at a cardiocenters, much fewer patients were referred by internal medicine physicians, cardiologists based at hospitals without cardiocenter and other medical specialities including general practitioners. Similarly, general practitioners first suspected the diagnosis of systemic amyloidosis only in 6% of cases [9]. Although these data demonstrate relatively satisfactory knowledge of clinical manifestations of CA, including ATTRwt, among Czech outpatient cardiologists working in private practice, the awareness of the disease appears to be insufficient among cardiologists based in smaller hospitals, internists and primary care physicians. A key task for shortening the whole diagnostic process is therefore to further increase awareness of main cardiac but also extracardiac manifestations of ATTRwt in these medical specialties, together with an emphasis on the possibility of non-invasive diagnostics and specific treatment of this disease.

Breathlessness together with other symptoms and signs of HF still represent the most common initial clinical profile of our patients. Similarly to our data, heart failure was found to be the main diagnostic pathway leading to ATTRwt-CA diagnosis in several other studies, prompting the final diagnosis in 44% to 79% cases [5, 8, 11–13]. Obviously, ATTRwt-CA must be part of differential diagnosis of HF symptoms in elderly patients.

Importantly, in almost 13% of our patients, the CA was first suspected based on the presence of suspicious echocardiographic findings in asymptomatic subjects with LV wall thickening. In accordance with our results, Italian authors reported in recent DIAMOND study that incidental imaging findings represented the diagnostic pathway leading to ATTRwt-CA diagnosis in one quarter of their patients, when 74% of these imaging studies were echocardiographic examinations [10]. These results underscore the importance of knowledge of so called echocardiographic “red flags” of CA when cardiac ultrasound shows LV wall thickening even in patients not suffering from HF [14]. Similarly, amyloid heart disease, especially ATTRwt, always must be considered in differential diagnosis of hypertrophic cardiomyopathy, especially in older patients [15].

Interestingly, abnormal result of DPD scintigraphy performed from non-cardiac indications was present as a main clinical scenario leading to suspicion of CA in almost 7% of our patients. Almost the same observation was reported by López-Sainz et al., who described incidental scintigraphy findings as clinical presentation prompting the diagnosis in 6% of their cohort [12]. In the study of González-López et al., 11% of ATTRwt patients were also diagnosed as an incidental finding after a positive DPD scan requested for oncologic or rheumatologic reasons [11]. These observations of us and others document adequate nuclear medicine specialists´ knowledge in searching for and interpreting incidental cardiac findings when evaluating bone scintigraphy using technetium-99 m-labeled diphosphonates.

However, our data show that several cardiac manifestations, that often occur in ATTRwt-CA, were the main reasons leading to suspicion of CA only in a small proportion of our patients. Atrial fibrillation or atrial flutter prompted the suspicion in less than 7% subjects and the presence of bradyarrhythmia requiring permanent pacemaker even in only 2.5%. This observation is in contrast with findings reported from the Spanish referral center, where atrial fibrillation led to diagnosis in 21% and conduction disorder in 6% of patients, respectively [12]. Similarly, González-López et al. reported that symptomatic atrioventricular block requiring a pacemaker prompted the diagnosis of CA in 7% of ATTRwt patients [11]. Moreover, atrial fibrillation (or atrial flutter) and permanent pacemaker implanted due to bradyarrhythmia had been present for several dozen months in one half and in one sixth of our subjects, respectively, before suspicion of CA was raised. Almost the same data were reported from Spanish referral center, where 50% of their ATTR patients had a history of atrial fibrillation and 21% were implanted with pacemaker [12]. A recent Italian DIAMOND study also showed, that 62% of their ATTR-wt patients suffered from atrial fibrillation and 15% were already implanted with a pacemaker [10]. It is therefore obvious that there is an evident need to increase awareness of the causal relationship between ATTRwt-CA and these rhythm disorders among physicians, which will definitely subsequently lead to reduction in diagnostic delay. The echocardiographic finding of LV wall thickening in elderly patients suffering from atrial fibrillation or conduction disorders should always arouse suspicion of ATTRwt, especially in the presence of other cardiac or extracardiac “red flags” of this disease.

The results of our survey also confirm the findings of previously published studies that have shown the frequent presence of some extracardiac pathologies which are typically expressed in ATTRwt individuals. First of all, the history of carpal tunnel syndrome was present in slightly more than a half of our patients. Almost the same prevalence of this feature of 56% was reported by Karam et al. in US-patients with ATTRwt [13]. Lesser frequency of carpal tunnel syndrome in ATTRwt subjects was described by López-Sainz et al., who found its history in 41% of their subjects [12]. The median duration of carpal tunnel syndrome history in our cohort was 8.3 years, which is completely in agreement with other finding showing that it can present 5 to 10 years prior to symptoms of ATTRwt-CA [16]. Other extracardiac manifestations of ATTRwt including polyneuropathy, lumbar spinal stenosis and biceps tendon rupture were found in a much smaller percentage of our patients.

At the time of the first examination at the dedicated center for ATTR-CA, two thirds of our patients were in NYHA class I or II and half of them were labelled NAC stage I. Other authors refer similar findings in their publications, in which they state that the percentage of individuals in NYHA classes I and II at the time of diagnosis reaches about 70% [5, 10]. We consider the fact that most of the patients are reported in less advanced stages of the disease to be extremely important. Low NYHA classes and NAC stage at the time of diagnosis are associated with much better life expectancy [17]. Consequently, establishing the diagnosis in the early stage of the disease allows to start specific therapy in a significant proportion of affected individuals. It is evident that only treatment of patients who are not in advanced stage of ATTRwt-CA is associated with a significant positive effect of disease-modifying therapy on mortality [18]. We believe that some echocardiographic data obtained during the first visit at the ATTR center also reflect the fact, that most of our patients were diagnosed at less advanced phase of ATTRwt-CA. Despite the Doppler indices of increased LV filling pressures were present in almost 60% cases, pulmonary hypertension was found only in approximately one third and increased right atrial pressure even only in less than one quarter of our patients. From a diagnostic point of view, it is important to emphasize that asymmetrical septal hypertrophy was seen in 5% of our patients. This is much less than reported by others, who found asymmetrical septal hypertrophy based on echocardiography or cardiac magnetic resonance in 23% and 79% ATTRwt individuals, respectively [11, 19]. Nevertheless, these findings clearly indicate that symmetrical (i.e. concentric) LV wall thickening does not represent a uniform expression of CA and asymmetrical septal hypertrophy must be considered in the differential diagnosis of amyloid heart disease. Furthermore, decreased LV ejection fraction (less than 50%) was described in almost one third of our patients. Very similar findings were published by Spanish authors, in whose study LV ejection fraction < 50% was seen in 36.8% individuals [11]. As ATTRwt-CA is very often cited as one of the causes of HFPEF in elderly [20], we would like to emphasize that decreased LV ejection fraction occurs relatively frequently in ATTRwt patients and CA must be taken into account also in clinical scenario of heart failure with reduced ejection fraction when echocardiography also demonstrates LV wall thickening [21].

Regarding ECG finding at the time of the first examination at the ATTR-CA center, we would like to emphasize, in addition to atrial fibrillation, the high incidence of conduction disorders, especially first-degree AV block and LAFB. Conduction system disease is common in ATTRwt -CA [22] and its presence in an old patient with LV wall thickening must be considered as a significant ECG “red-flag” that should lead to suspicion of CA. In comparison, low QRS voltage were present only in about one tenth of our ATTRwt patients. It is even a smaller detection rate, then reported by González-López et al., who found low RRS voltages in 22% of their cohort [11]. On the other hand, ECG voltage criteria for LV hypertrophy were seen in only 2 of our patients showing that the mismatch between LV wall thickening detected by imaging methods and the absence of ECG signs of LV hypertrophy represents very strong indicator of the possible presence of CA [6].

The definite ATTRwt-CA diagnosis was made noninvasively in almost two thirds of our patients. Nevertheless, invasive diagnostics based on endomyocardial biopsy was still necessary in 37% individuals, mainly due to abnormalities in laboratory values of free light chain ratios or serum immunoelectrophoresis. Importantly, we did not find any significant difference in the length of the whole diagnostic process since the suspicion of CA to its final confirmation between noninvasive and invasive pathways. In a recent study from Spanish referral center, endomyocardial biopsy was only required in 22% of their patients [12]. These results clearly show that noninvasive diagnostics, based on hematological laboratory tests and scintigraphy, has become the main method for identifying ATTRwt-CA in the last decade. For comparison, in 2017 González-López et al. reported that a non-invasive diagnosis was made in only 38% of their cohort [11]. Although non-invasive diagnostic pathway is currently possible in most ATTRwt patients, endomyocardial biopsy is still necessary in a certain proportion of subjects. This fact represents one the reasons why it is necessary to concentrate individuals with suspected CA in dedicated specialized centers where endomyocardial biopsy is routinely and safely performed.

## Study limitations

We are well aware of the main limitation of our survey which is associated with its time-limited cross-sectional character. However, we believe that our cohort is representative sample of ATTRwt-CA patients diagnosed in the Czech Republic in the current era. Important limitation is also given by the fact, that information in the dedicated questionnaire was provided retrospectively by elderly patients with multimorbidity. The retrospective symptom attribution is particularly challenging in these individuals whose possible age-related memory deficits could bias the validity of their responses.

We also acknowledge that in many cardiac and extracardiac symptoms and pathologies the extent to which they are directly attributable to ATTRwt is uncertain. However, the character of our survey does not enable to ascertain the full aetiology of the reported manifestations.

## Conclusions

Despite a short time interval between the suspicion of CA and definite ATTRwt-CA diagnosis, which is given by the wide availability of DPD scintigraphy, there is still relatively unsatisfactory delay between the onset of clinical manifestations and suspicion of CA reaching more than a year. Heart failure is the most common clinical profile leading to suspicion of CA, however other scenarios are not rare. To shorten the whole diagnostic process of ATTRwt-CA in the Czech Republic, it is necessary to increase awareness of the disease and related cardiac and extracardiac manifestations that may precede main diagnostic symptoms by even several tens of months, especially among cardiologists based in regional hospitals, internists and general practitioners. Importantly, most ATTRwt patients are currently diagnosed noninvasively and in the early stages of the disease, which allows to use specific therapy improving both mortality and morbidity in a significant part of individuals.

## Supplementary Information

Below is the link to the electronic supplementary material.

Supplementary Material 1

## Data Availability

All data generated or analysed during this study are included in this published article (and its supplementary information files).

## References

[1] Kittleson MM, Maurer MS, Ambardekar AV, Bullock-Palmer RP, Chang PP, Eisen HJ, American Heart Association Heart Failure and Transplantation Committee of the Council on Clinical Cardiology, et al. Cardiac amyloidosis: evolving diagnosis and management: A scientific statement from the American heart association. Circulation. 2020;142(1):e7–22. 10.1161/CIR.0000000000000792. 32476490 10.1161/CIR.0000000000000792

[2] Gonzalez-Lopez E, Maurer MS, Garcia-Pavia P. Transthyretin amyloid cardiomyopathy: a paradigm for advancing precision medicine. Eur Heart J. 2025;46:999–1013. 10.1093/eurheartj/ehae811. 39791537 10.1093/eurheartj/ehae811 PMC11905746

[3] Fontana M, Berk JL, Drachman B, Garcia-Pavia P, Hanna M, Lairez O, et al. Changing treatment landscape in transthyretin cardiac amyloidosis. Circ Heart Fail. 2025;18(8):e012112. 10.1161/CIRCHEARTFAILURE.124.012112. 40160093 10.1161/CIRCHEARTFAILURE.124.012112 PMC12356574

[4] Lane T, Fontana M, Martinez-Naharro A, Quarta CC, Whelan CJ, Petrie A, et al. Natural History, quality of Life, and outcome in cardiac transthyretin amyloidosis. Circulation. 2019;140:16–26. 10.1161/CIRCULATIONAHA.118.038169. 31109193 10.1161/CIRCULATIONAHA.118.038169

[5] Ladefoged B, Dybro A, Povlsen JA, Vase H, Clemmensen TS, Poulsen SH. Diagnostic delay in wild type transthyretin cardiac amyloidosis - A clinical challenge. Int J Cardiol. 2020;304:138–43. 10.1016/j.ijcard.2019.12.063. 32033783 10.1016/j.ijcard.2019.12.063

[6] Garcia-Pavia P, Rapezzi C, Adler Y, Arad M, Basso C, Brucato A, et al. Diagnosis and treatment of cardiac amyloidosis. A position statement of the European society of cardiology working group on myocardial and pericardial diseases. Eur J Heart Fail. 2021;23:512–26. 10.1002/ejhf.2140. 33826207 10.1002/ejhf.2140

[7] Dang D, Fournier P, Cariou E, Huart A, Ribes D, Cintas P, et al. Gateway and journey of patients with cardiac amyloidosis. ESC Heart Fail. 2020;7:2418–30. 10.1002/ehf2.12793. 32588554 10.1002/ehf2.12793 PMC7524246

[8] Ihne-Schubert SM, Leberzammer M, Weidgans M, Frantz S, Einsele H, Knop S, et al. Single German centre experience with patient journey and care-relevant needs in amyloidosis: the German AMY-NEEDS research and care program. PLoS ONE. 2024;19:e0297182. 10.1371/journal.pone.0297182. 38768126 10.1371/journal.pone.0297182 PMC11104610

[9] Damy T, Adams D, Bridoux F, Grateau G, Planté-Bordeneuve V, Ghiron Y, et al. Amyloidosis from the patient perspective: the French daily impact of amyloidosis study. Amyloid. 2022;29:165–74. 10.1080/13506129.2022.2035354. 35144512 10.1080/13506129.2022.2035354

[10] Tini G, Milani P, Zampieri M, Caponetti AG, Fabris F, Foli A, et al. Diagnostic pathways to wild-type transthyretin amyloid cardiomyopathy: a multicentre network study. Eur J Heart Fail. 2023;25:845–53. 10.1002/ejhf.2823. 36907828 10.1002/ejhf.2823

[11] González-López E, Gagliardi C, Dominguez F, Quarta CC, de Haro-Del Moral FJ, Milandri A, et al. Clinical characteristics of wild-type transthyretin cardiac amyloidosis: disproving Myths. Eur Heart J. 2017;38:1895–904. 10.1093/eurheartj/ehx043. 28329248 10.1093/eurheartj/ehx043

[12] López-Sainz Á, Hernandez-Hernandez A, Gonzalez-Lopez E, Domínguez F, Restrepo-Cordoba MA, et al. Clinical profile and outcome of cardiac amyloidosis in a Spanish referral center. Rev Esp Cardiol. 2021;74:149–58. 10.1016/j.rec.2019.12.020. 32317158 10.1016/j.rec.2019.12.020

[13] Karam C, Moffit C, Summers C, Merkel MP, Kochman FM, Weijers L, et al. The journey to diagnosis of wild-type transthyretin-mediated (ATTRwt) amyloidosis: a path with multisystem involvement. Orphanet J Rare Dis. 2024;19:419. 10.1186/s13023-024-03407-3. 39516862 10.1186/s13023-024-03407-3 PMC11549766

[14] Jurcuţ R, Onciul S, Adam R, Stan C, Coriu D, Rapezzi C, et al. Multimodality imaging in cardiac amyloidosis: a primer for cardiologists. Eur Heart J Cardiovasc Imaging. 2020;21:833–44. 10.1093/ehjci/jeaa063. 32393965 10.1093/ehjci/jeaa063

[15] Garcia-Pavia P, Damy T, Piriou N, Barriales-Villa R, Cappelli F, Bahus C, et al. Prevalence and characteristics of transthyretin amyloid cardiomyopathy in hypertrophic cardiomyopathy. ESC Heart Fail. 2024;11:4314–24. 10.1002/ehf2.14971. 39210606 10.1002/ehf2.14971 PMC11631301

[16] Wininger AE, Phelps BM, Le JT, Harris JD, Trachtenberg BH, Liberman SR. Musculoskeletal pathology as an early warning sign of systemic amyloidosis: a systematic review of amyloid deposition and orthopedic surgery. BMC Musculoskelet Disord. 2021;22:51. 10.1186/s12891-020-03912-z. 33419417 10.1186/s12891-020-03912-z PMC7796584

[17] Gillmore JD, Damy T, Fontana M, Hutchinson M, Lachmann HJ, Martinez-Naharro A, et al. A new staging system for cardiac transthyretin amyloidosis. Eur Heart J. 2017;39:2799–806. 10.1093/eurheartj/ehx589. 10.1093/eurheartj/ehx589 29048471

[18] Maurer MS, Schwartz JH, Gundapaneni B, Elliott PM, Merlini G, Waddington-Cruz M, et al. Tafamidis treatment for patients with transthyretin amyloid cardiomyopathy. N Engl J Med. 2018;379:1007–16. 10.1056/NEJMoa1805689. 30145929 10.1056/NEJMoa1805689

[19] Martinez-Naharro A, Treibel TA, Abdel-Gadir A, Bulluck H, Zumbo G, Knight DS, et al. Magnetic resonance in transthyretin cardiac amyloidosis. J Am Coll Cardiol. 2017;70:466–77. 10.1016/j.jacc.2018.03.536. 28728692 10.1016/j.jacc.2017.05.053

[20] Magdi M, Mostafa MR, Abusnina W, Al-Abdouh A, Doss R, Mohamed S, et al. A systematic review and meta-analysis of the prevalence of transthyretin amyloidosis in heart failure with preserved ejection fraction. Am J Cardiovasc Dis. 2022;12:102–11. 35873185 PMC9301026

[21] Achten A, Muller SA, Wijk SS, van der Meer MG, van der Harst P, et al. Diversity of heart failure phenotypes in transthyretin amyloid cardiomyopathy. More than just heart failure with preserved ejection fraction. Ann Med. 2024;56:2418965. 10.1080/07853890.2024.2418965. 39460551 10.1080/07853890.2024.2418965 PMC11514392

[22] Donnellan E, Wazni OM, Saliba WI, Hanna M, Kanj M, Patel DR, et al. Prevalence, Incidence, and impact on mortality of conduction system disease in transthyretin cardiac amyloidosis. Am J Cardiol. 2020;128:140–6. 10.1016/j.amjcard.2020.05.021. 32650908 10.1016/j.amjcard.2020.05.021

