# Supplementary material for: Wild-type transthyretin cardiac amyloidosis: the journey to diagnosis in the Czech Republic: The research project of the Czech Society of Cardiology
Source: Orphanet J Rare Dis. 2026 Feb 13;21:103. doi: 10.1186/s13023-026-04249-x (PMC13005566; doi:10.1186/s13023-026-04249-x)
Supplement: Supplementary file 1 — Supplementary Material 1 [file 13023_2026_4249_MOESM1_ESM.pdf]

Questionnaire – ATTRwt-CA: Diagnostic Pathway

Patient code in e-CRF:

Sex: - male / - female

Age at time of ATTR diagnosis (years):

Status: - lives alone / - lives in cople/ - lives with siblings/ - nursing home–long-term care / - other

Children: - yes / - no

Residence:

- capital city / - city (>100,000 inhabitants) / - city (50–100,000) / - city (10–50,000) / - city (<10,000) / - village

First suspicion of ATTRwt raised by (only one option):

- general practitioner / - outpatient hematologist / - hospital hematologist / - outpatient neurologist / - hospital neurologist / - outpatient internist / - outpatient cardiologist / - hospital internist / - hospital cardiologist (non-cardiac center) / - hospital cardiologist (cardiac center) / - cardiologist in ATTR center / - CMR physician / - nuclear medicine specialist / - other physician

First symptom / finding leading to suspicion of ATTRwt (only one option):

- heart failure symptoms/ - chest pain / - stroke / - de-escalation of arterial hypertension or tendency to hypotension / - atrial fibrillation or flutter / - bradyarrhythmia requiring pacemaker / - low QRS voltage on ECG and/or pseudo-MI pattern / - asymptomatic conduction abnormality on ECG (sub-choice: unspecified / AV block / LAHB / RBBB / LBBB / nonspecific conduction disturbance) / - other ECG changes / - known aortic stenosis / - asymptomatic echocardiographic finding / - DPD scan from non-cardiac indication / - CMR finding / - asymptomatic BNP or NT-proBNP elevation / - asymptomatic troponin elevation / - known aortic stenosis / - history of carpal tunnel syndrome / - spinal stenosis / - spontaneous biceps rupture / - polyneuropathy / - history of dysautonomia / - hearing loss / - other / ¨

Time from first ATTR symptom/finding to suspicion of ATTR diagnosis: (months)

Time from first ATTR symptom/finding to final ATTR diagnosis: (months)

Were other typical ATTR symptoms/findings already present at the time of suspicion? - yes / - no

If yes, for how long (months): (multiple choice)

- heart failure symptoms/ - chest pain / - syncope / - stroke / - decapitation of arterial hypertension or tendency to hypotension / - low QRS voltage on ECG and/or pseudo-MI pattern / - atrial fibrillation or flutter / - asymptomatic conduction abnormality on ECG (sub-choice: unspecified / AV block / LAHB / RBBB / LBBB / nonspecific conduction disturbance) / - implanted pacemaker / - aortic stenosis / - history of carpal tunnel syndrome / - spinal stenosis / - spontaneous biceps rupture / - polyneuropathy / - history of dysautonomia / - hearing loss / - cannot be determined

Number of physicians visited from first ATTR symptom/finding until suspicion was raised:

- 1 / - 2 / - 3 / - 4 / - 5 or more

Any previous diagnosis / misdiagnosis? - yes / - no / - cannot be determined

If yes, which one:

- hypertensive heart disease / - HCM / - RCM / - HFpEF / - HFrEF / - aortic stenosis / - CAD / - other (AF/flutter, conduction disorder)

Who finally established ATTR diagnosis?

- general practitioner / - outpatient internist / - outpatient neurologist / - outpatient cardiologist / - hospital internist / - hospital neurologist / - hospital cardiologist (non-cardiac center) / - hospital cardiologist (cardiac center) / - cardiologist in ATTR center / - other physician

ATTR diagnosis established: - non-invasively / - invasively

If DPD scan performed, Perugini score:

NYHA class at time of ATTR diagnosis: I / II / III / IV

Medications at time of ATTR diagnosis:

- ACEi or ARB / - ARNI / - beta-blocker / - calcium antagonist / - diuretic / - MRA / - digoxin / - anticoagulant / - antiarrhythmic

NT-proBNP level at first visit in ATTR center:

eGFR at first visit in ATTR center: (ml/min)

ECG at first visit in ATTR center (multiple choice):

- sinus rhythm / - atrial fibrillation or flutter / - paced rhythm / - other basic rhythm / - AV block I / - AV block II Mobitz I / - AV block II Mobitz II / - complete AV block / - LAHB / - RBBB / - LBBB / - nonspecific conduction disturbance / - low QRS voltage / - LVH voltage criteria / - pseudo-MI pattern

Echocardiography at time of diagnosis in ATTR center:

- IVS (mm) / - posterior wall thickness (mm) / - LVEDD (mm) / - LVEF (%) / - s´ septal / - E/e´ septal / - LAVi (ml/m^2) / - RV dimension in A4C (mm) / - TAPSE (mm) / - estimated PASP (mmHg) / - estimated RA pressure (normal / borderline / high / not measurable) / - pericardial effusion (yes/no) / - aortic stenosis (yes/no)
